# Genetic and Environmental Influences on Female Sexual Orientation, Childhood Gender Typicality and Adult Gender Identity

**DOI:** 10.1371/journal.pone.0021982

**Published:** 2011-07-07

**Authors:** Andrea Burri, Lynn Cherkas, Timothy Spector, Qazi Rahman

**Affiliations:** 1 Biological and Experimental Psychology Group, School of Biological and Chemical Sciences, Queen Mary University of London, London, United Kingdom; 2 Department of Twin Research and Genetic Epidemiology, King's College London, London, United Kingdom; University of Hong Kong, Hong Kong

## Abstract

**Background:**

Human sexual orientation is influenced by genetic and non-shared environmental factors as are two important psychological correlates – childhood gender typicality (CGT) and adult gender identity (AGI). However, researchers have been unable to resolve the genetic and non-genetic components that contribute to the covariation between these traits, particularly in women.

**Methodology/Principal Findings:**

Here we performed a multivariate genetic analysis in a large sample of British female twins (*N* = 4,426) who completed a questionnaire assessing sexual attraction, CGT and AGI. Univariate genetic models indicated modest genetic influences on sexual attraction (25%), AGI (11%) and CGT (31%). For the multivariate analyses, a common pathway model best fitted the data.

**Conclusions/Significance:**

This indicated that a single latent variable influenced by a genetic component and common non-shared environmental component explained the association between the three traits but there was substantial measurement error. These findings highlight common developmental factors affecting differences in sexual orientation.

## Introduction

Understanding of the origins of sexual orientation can help narrow competing developmental explanations for behavioral sex differences in general and is of increasing importance to researchers concerned with the physical and mental health of sexual minorities [1], [2]. Homosexuality appears to be a stable sexual phenotype in humans with population-based surveys suggesting lifetime prevalence of 2–4% in men and 0.5–1.5% in women when measured as exclusive same-sex “feelings” (e.g., homosexual attractions and fantasies) [3], [4]. The distribution of the trait is generally bimodal and this is stronger for men than it is for women; a first indication of different, albeit overlapping, developmental pathways towards male versus female sexual orientation [5], [6]. Basic biobehavioral research into female sexual orientation appears infrequent compared to that performed on males.

Several early family and twin studies provide evidence for a genetic component to both male and female sexual orientation [7], [8], [9]. Heritability estimates were found to be in the region of 40% to 50%. However, the putative inheritance patterns have remained unclear. Family pedigree studies in men have suggested that maternally inherited factors might be involved [10], [11], [12], [13] although one large study in a carefully ascertained pedigree has failed to replicate this [14]. Among females, autosomal and sex-linked routes have been implicated although there has only ever been one survey study performed here [15]. Two preliminary linkage studies reported microsatellite marker loci for male homosexuality on the X chromosome [10], [11] with one confirming linkage for males but not females [11]. However, two independent reports found no such linkage in males [16], [17]. The latest genome-wide scan reported several new autosomal markers for male sexual orientation [18] which again require replication.

Early criticisms of previous studies focused on the possibility that their reliance on self-selected volunteers (e.g., through advertisements in gay and lesbian press) may have biased the results by increasing twin resemblance. But it is not clear how this would inflate concordance rates or overestimate genetic or non-genetic effects (similarity would be increased for both MZ and DZ twins). However, two studies of attraction components of sexual orientation, and one of same-sex sexual behavior, were population-based and all reported lower concordance rates than previously found at around 30% - although Bailey et al. 2000 were unable to resolve genetic, shared and non-shared environmental factors in their univariate models [19], [20], [21]. One of these reports supported the notion that developmental pathways towards homosexuality might be different for men and women [19]. One further study which modeled several components of sexual orientation (attractions, attitudes to homosexual sex, and lifetime same-sex partners) reported stronger evidence for genetic influences of between 50% and 60% in females and approximately 30% in males [22]. Despite the inconsistency of findings across these studies in terms of the magnitude of the heritability estimates, all of them suggest a genetic component to sexual orientation.

Sexual orientation, like many complex behaviors, comes as a “package” of covarying traits. Critical among these are childhood gender typicality (or CGT), which are sex-typed behaviors, activities and interests that are statistically atypical for biological sex during childhood) and gender identity (psychological gender as “masculine” or “feminine” during adulthood). CGT is robustly correlated with adult homosexuality as demonstrated in prospective and retrospective studies and has been observed cross-culturally [9], [23], [24], [25]. In order to control for possible retrospective memory biases (based on the argument that homosexuals might exaggerate nontypicality and heterosexuals understate it) one study has confirmed the association between CGT and homosexuality using home videos of childhood behavior [26]. Adult gender identity (AGI), although not a psychometric homologue to CGT, also shows an association with sexual orientation as measured via ratings of self-ascribed masculine or feminine feelings, traditional personality measures of gender (e.g., the Bem Sex Role Inventory) and occupational interests [27], [28]. Twin models show that both CGT and AGI are heritable although the estimates vary. Knafo et al. [29] reported heritability estimates of 37% in boys and almost 80% in girls (3–4 year olds). Iervolino et al. [30] examined the full range of normal variation in CGT in the same dataset and found 34% heritability in boys compared to 57% in girls. Van Beijsterveldt et al. [31] reported 70% for CGT in both sexes among 7 and 10-year old twins. Bailey et al. [19] reported heritability of 50% for men and 37% for women in retrospectively recalled CGT. A similar wide range of estimates applies to twin models of gender identity. Lippa and Hershberger [32] reported modest heritability for their measure based on occupational interests at 53% (no sex differences) whereas Bailey et al. [19] reported estimates of 31% for men and 24% for women. Finally, Bailey et al. [19] reported that the covariation between sexual orientation, CGT and AGI could be explained by a common familial factor although the model was a poor statistical fit.

Taken together the data suggest that CGT (and to a lesser extent, AGI) might be considered as a possible “sex atypicality” endophenotype for trait sexual orientation which could be more powerful in future gene discovery. Trait sexual orientation is notoriously skewed. Thus, a broad research strategy which includes CGT and AGI with their more favorable statistical distributions will enhance our ability to resolve the molecular genetics of sexuality.

A related conceptual issue concerns the putative etiological factor(s) which explain the link between CGT, AGI and sexual orientation and thus may constitute the “sex atypicality” endophenotype. A good candidate is prenatal sexual differentiation under the action of androgens [1], [33]. Homosexuals are viewed as having been subject to atypical levels of prenatal androgens thus causing sex-atypical differentiation of brain structures that control direction of sexual preference, gender-related psychological traits (including CGT and AGI) and related traits (such as specific cognitive differences). However, there are very few direct tests of this hormonal link between measures of sex-atypicality and trait sexual orientation. There are also few tests of the developmental progression of this process. For example, do genetic variations contribute to atypical hormonal levels which shape CGT and then does CGT precedes the development of trait sexual orientation? Or are CGT and sexual orientation simply correlated but develop independently? Strong evidence for an association between prenatal androgen levels, CGT and sexual orientation comes from studies of women with congenital adrenal hyperplasia (CAH) who have been exposed to high levels of prenatal adrenal androgens. Girls (and adolescents) with CAH show sex-typed behavior and interests in the male-typical direction, in spite of strong sex-typical parental gender socialization [34], [35], [36]. Adult females with CAH also report significantly more bisexual/homosexual fantasies and attractions relative to their control sisters [37], [38], [39]. Prospective studies in non-clinical populations also suggest that variations in fetal levels of testosterone (measured via amniotic sampling) are associated with male-typical gender related behaviors in girls [40], [41]. Finally, a meta-analysis of the relationship between sexual orientation and the ratio of the 2^nd^ to 4^th^ finger digits (a somatic marker ascribed to the prenatal actions of androgen exposure) revealed a significant association between male-typical digit ratios and sexual orientation in women [42]. These lines of evidence support the notion of a developmental coupling between levels of prenatal androgen, gender-related behaviors and interests, and sexual orientation among women.

In the present study, we analyzed questionnaire data from a large volunteer register of female twins in the United Kingdom to test the hypotheses that (1) genetic factors significantly influence variation in measures of sexual orientation and it's two covariates – CGT and AGI; (2) that these three traits correlate significantly at the phenotypic level; and (3) that the covariation among the three traits is also due to a genetic correlation. This is the first study of its kind in a British sample.

## Methods

### Ethic statement

The study was approved by the St. Thomas' Hospital research ethics committee. All study participants involved in this study provided informed written consent.

### Participants and questionnaire

Subjects were monozygotic (MZ) and dizygotic (DZ) volunteer female twins drawn from the “TwinsUK” registry [43]. Due to unavailability of data, no males were included in this study. Zygosity was established using standardized questions about physical similarity and confirmed by multiplex DNA genotyping in cases of uncertainty [44].

In 2002, twins were sent a questionnaire asking about general sexual behavior and sexual orientation (referring to “sexual attractions” with men and women in this study). Of the 8,418 questionnaires sent, 4,725 (56.1%) were returned. In a 2005 follow-up survey, an anonymous questionnaire assessing CGT and AGI was also sent to 6,934 female twins in the registry and returned by 4,850 (69.9%). The questionnaires were developed previously based on scales in the published literature but shortened for the purposes of practicality within a large twin register. Twins were not selected on the basis of variables being studied and were unaware of any hypothesis being tested.

Final questionnaire data relating to sexual orientation and its psychological correlates, CGT and AGI, was available on a total of 4,426 female twin individuals - a 49% response rate. Females who reported never having felt sexually attracted to anyone else (*N* = 44; 0.99%) and/or reported never having had sexual experiences (*N* = 51; 1.15%) were excluded from the analyses as were 228 (5.15%) females with missing values for any items assessing CGT and AGI. Also, 32 (0.72%) women were excluded because of unknown zygosity. After applying exclusion criteria, a total of 4,066 women were eligible for analysis, comprising 906 complete MZ pairs, 806 complete DZ pairs and 642 women whose co-twins did not participate (15.35%). However, sample sizes varied somewhat in the different analyses because of missing scale data.

Demographic information on all twins including age, marital status, and years of education were obtained from the TwinsUK database.

### Measures

#### Childhood gender typicality (CGT) and adult gender identity (AGI)

The CGT scale consisted of four items retrospectively assessing childhood sex-typed behavior and gender identity which are comparable to several published scales [19], [45]. Example items include “As a child I was called a ‘tomboy’ by my peers” and “As a child I preferred playing with boys rather than girls”. Assessment of participants' self-concepts as masculine or feminine (AGI) was computed using four items comparable to those used by Bailey et al. [19]. Example items include “I don't feel very masculine” and “I pride myself on being feminine”. Scores for CGT and AGI were derived by adding the point values for each of the four scale-specific items together and dividing it by the number of scale items. Response options were on a 7-point Likert-type scale, ranging from “strongly agree” (1) to “strongly disagree” (7). Cronbach's alpha, a measure of internal consistency, was 0.62 for CGT and 0.44 for AGI. High scores on each measure mean more feminine.

#### Sexual orientation

Sexual orientation was measured using a scale, similar in kind to Kinsey-type scales used extensively in sexuality research assessing sexual attraction (degree of attraction towards the same or opposite sex. Response options for this measure ranged from 1 (“only to/with males, never to/with females”) to 5 (“only to/with females, never to/with males”) with a supplementary option of “no sexual attraction” (numbered 6) [19], [46].

### Analysis

For descriptive and genetic analysis CGT, AGI and sexual attraction were treated as continuous traits based on women's responses to the specific questions. Unpaired *t*-tests (two-tailed) were used to examine differences between MZ and DZ twins on age, years of education, CGT, AGI and mean sexual orientation scores. Two-sample tests of proportions were used to test for differences in marital status and SES. Pearson's correlation coefficients were used to explore patterns of association between CGT, AGI and sexual orientation scores.

For all analyses, a *P* value less than 0.05 or odds ratios with a 95% confidence interval not including “1” were considered statistically significant, unless stated otherwise. Data handling and descriptive analyses were undertaken using STATA (Intercooled Stata for Windows 95, Version 5.0, 1997, StataCorp, College Station, TX) while all genetic modelling was carried out with Mx software [47].

#### Univariate genetic modelling

The present study used the classical twin design where population variance in phenotypes, as well as covariance between them, can be dissected into genetic and environmental sources. The twin design assumes that MZ twins share 100% of both their genes and shared environment, whereas DZ twins share - on average - 50% of their genes and 100% of shared environment. Presuming that both types of twins share equally similar family environments, any greater similarity between MZ as compared with DZ twin pairs is attributed to genetic factors.

In the present study maximum likelihood genetic modeling was used to model latent genetic and environmental factors influencing sibling covariance in CGT, AGI and sexual orientation for MZ and DZ twins. Bivariate normality was given for the measures of CGT and AGI after the variables were transformed. However, for trait such as sexual orientation, normality cannot be achieved.

Genetic model fitting was used to decompose the observed phenotypic variance (P) into additive (A) and dominant (D) genetic effects, and shared (C) and non-shared environmental (E) effects [48]. The shared environmental variance refers to factors shared between twin pairs such as family environment. The non-shared environmental variance reflects factors affecting each twin individually (e.g., specific prenatal events or peer socialization) and also includes measurement error.

For continuous phenotypes, evidence for a genetic contribution (heritability or *h* ^2^) can be obtained by comparing similarities in scores using intra-class correlation coefficients (ICCs) for MZ and DZ twin pairs. Depending on the correlations between the MZ and DZ twins, either an ACE or an ADE model is fitted. For univariate models in the present study, an ACE model was applied when DZ correlations were more than half the MZ correlations. When the DZ correlations were less than half the MZ correlations, both ACE and ADE were estimated for comparative purposes [49]. Initial assessment of the components (A, D, C, and E) may suggest non-significant values in one or more component. Further analysis can determine the significance of each factor as components of the observed variance by removing each sequentially from the full model and testing the deterioration in fit of the various nested models, using the likelihood ratio test. In the present study, the fit of the different models was compared by taking the fit function and the degrees of freedom (*df*) of the full model and subtracting it from the fit function and the *df* of the nested restricted models. The subtraction gives an *χ2* value and associated *df* that can be tested for significance. In addition, the Akaike Information Criteria (AIC = *χ2*-2df) was considered, with lower values indicating the most suitable model. The most parsimonious model was then used to estimate the heritability. Note the assumption that trait-related environments are similar to the same degree in MZ and DZ pairs is valid for trait sexual orientation (Bailey et al., 2000; Kendler et al., 2000). More detailed descriptions of twin modeling analyses can be found in Posthuma et al. [50].

#### Multivariate genetic modelling

Using cross-twin and cross-trait correlations allows us to partition the covariance between traits into genetic and environmental components and therefore permit the quantification of any overlap in the genetic or environmental correlation between traits. Here we present both the estimated genetic covariance between the traits as a proportion of the total phenotypic covariance (bivariate heritability) and the proportion of the total genetic variance for the traits (genetic correlation).To test our hypothesis that the covariation among CGT, AGI, sexual orientation (attraction) can be explained by genetic correlation between the traits, we further fitted the following three multivariate models to the data [48], [51]: (1) The Cholesky decomposition provides the correlations between the three independent genetic and environmental factors (A, C, D, E) and decomposes the variance for a trait into additive and non-additive genetic and non-shared environmental effects, providing the fullest potential explanation of the data. (2) The independent pathway model is a submodel of the Cholesky model and tests whether covariance between the traits is explained by a single underlying genetic factor and a single underlying environmental factor. (3) The common pathway model assumes that a single shared latent factor underlies all three measures.

The suitability of the multivariate models was determined by comparing the models AIC, BIC (Bayesian Information Criterion) and their goodness of fit as measured with the likelihood ratio chi-square test (−2LL).

## Results

### Descriptive analysis

Table 1 displays the participant characteristics for the whole sample and by zygosity group. The MZ and DZ twin groups were well matched for sexual orientation, CGT, AGI and most demographic variables except for marital status where MZ twins were significantly more often married compared with DZ twins (44.32% vs. 39.10%; *P*<0.01). Also DZ twins, more than MZ twins, reported being in a relationship (35.82% vs. 39.85%; *P*<0.05) or being widowed (5.58% vs. 7.36%; *P*<0.01).

**Table 1:** Means (and standard deviations) for continuous demographic variables, CGT, AGI and sexual orientation (attraction), along with frequency data for discrete demographics for the whole sample and by zygosity group.

	Overall (*N* = 4,066)		MZ (*N* = 2,098)		DZ (*N* = 1,998)		P-value*
	Mean (SD)	Range	Mean (SD)	Range	Mean (SD)	Range	
Age	53.36 (12.65)	16–87	53.10 (13.43)	16–87	53.66 (11.73)	16–81	0.11
Education in years	10.40 (2.91)	3–33	10.50 (2.93)	6–33	10.35 (2.88)	3–32	0.09
CGT	5.22 (1.25)	1–7	5.25 (1.25)	1–7	5.19 (1.26)	1–7	0.12
AGI	4.39 (0.89)	1–7	4.38 (0.89)	1–7	4.41 (0.91)	1–7	0.28
Sexual attraction	1.13 (0.46)	1–5	1.12 (0.41)	1–5	1.14 (0.50)	1–5	0.16

	*N*	%	*N*	%	*N*	%	P-value**
Marital status							
Single	209	7.29	96	6.86	113	7.70	0.30
Married	1,194	41.65	620	44.32	574	39.10	0.00
In relationship	1086	37.88	501	35.82	585	39.85	0.01
Divorced	212	7.39	104	7.43	108	5.99	0.07
Widowed	166	5.79	78	5.58	88	7.36	0.02

*Unpaired two-tailed *t*-test and Mann-Whitney U-tests were used to test for mean differences in response frequencies.

**Two-sample test of proportions were used to explore differences in response frequencies.

Most women displayed “average” AGI scores with the peak score being at 4.25 (30.9% of total sample). A small fraction of women had values at both extreme ends of the distribution. Overall, AGI showed less variability compared with CGT. Whilst only a negligible proportion of subjects reported a high degree of childhood gender nonconforming behavior, most of the women scored in the upper third of the distribution, with peak scores at 5.5 and 7. We also observed the previously documented general tendency for women to show more non-heterosexuality at the predominantly heterosexual end of the two scales (see Table 2).

**Table 2:** Percentage of women that checked each item of sexual attraction along with means (and standard deviations) for their respective CGT and AGI scores.

Measure: “I have felt sexually *attracted*”…”		% Sexual attraction	CGT mean score (SD)	AGI mean score (SD)
1	Only to/with males, never to/with females	89.92	5.98 (8.01)	4.43 (0.88)
2	More to/with males than females	8.56	5.44 (8.74)	4.22 (0.91)
3	Equally to/with males and females	0.29	3.90 (1.57)	4.53 (0.55)
4	More to/with females than males	0.86	3.89 (1.33)	4.33 (0.99)
5	Only to/with females, never to/with males	0.36	4.12 (1.41)	4.15 (0.65)

### Twin similarity

Intra-class correlations for MZ and DZ twins in the three measures are reported in Table 3. For all measures MZ twin correlations were consistently higher compared with DZ twin correlations, indicating a genetic contribution to the variance in these traits. However, the correlations were also all modest indicating a substantial influence of non-shared environmental factors. In the case of AGI the correlations were very low militating against a genetic contribution. For all traits, except AGI, DZ correlations were less than half the MZ correlations pointing to the involvement of dominant genetic effects (Table 3).

**Table 3:** Intra-class correlations, cross-twin cross-trait correlations and heritabilities for CGT, AGI and both measures of sexual orientation.

	CGT twin 1	AGI twin1	Sexual attraction twin1	Heritability % (95% CI)
CGT twin 2	0.36/0.02	0.03	−0.02	0.32 (0.26–0.37)
AGI twin 2	0.03	0.11/0.07	−0.02	0.11 (0.05–0.17)
Sexual attraction twin2	−0.13	−0.06	0.28/0.04	0.25 (0.17–0.33)

Heritability estimates and 95% CIs for all variables are calculated from the best-fitting, most parsimonious univariate AE model.

Note. Twin correlations for MZs/DZs are presented on the diagonal. Cross-twin cross-trait correlations for MZs are presented below the diagonal. Cross-twin cross-trait correlations for DZs are presented above the diagonal.

### Univariate model fitting

Based on the intra-class correlations, ACE and ADE models were fitted for all phenotypes. For all measures the best-fitting model was an AE model (Table 3). The highest heritability was found for CGT (32%). Heritability was moderate for sexual attraction (25%) and small for AGI (11%). There was no effect of dominance on any of the measures. There was a larger contribution of non-shared environmental factors to AGI than to CGT. In contrast to previous studies which produced relatively large confidence intervals [19], [20], [21], our confidence intervals were relatively narrow in comparison (see Table 3) although probably larger than other twin studies of psychological individual differences traits (such as personality). Nevertheless, they suggest some stability of our point estimates.

### Multivariate model fitting

Genetic and environmental correlations derived from the ADE Cholesky model are shown in Table 4, along with the phenotypic correlations. We found significant associations between all three measures; hence, all measures were included in the multivariate analyses. The significant correlations ranged from *r* = −0.21 to *r* = 0.05, with the highest correlation being between CGT and sexual attraction and the lowest between AGI and sexual attraction (Table 4). The Cholesky results indicated that a considerable degree of genetic correlation exists especially between sexual attraction and CGT and AGI (r = −0.42 and r = −0.45, respectively). The bivariate heritability suggested that approximately 57% of the covariance between CGT and sexual attraction is due to additive genetic factors with the remaining 43% attributable to unique environmental effects (Table 4).

**Table 4:** Phenotypic, genetic and non-shared environmental correlations among CGT, AGI and sexual orientation (attraction).

	CGT-AGI	CGT-Sexual attraction	AGI-Sexual attraction
r_p_	0.12	−0.21	−0.06
proportion of r_P_ due to:			
A	0.27	0.57	0.11
D	0.05	0.00	0.00
E	0.68	0.43	0.89
Correlations:			
*r*_A_	0.21	−0.42	−0.45
*r*_D_	-	-	-
*r*_E_	−0.11	−0.13	0.00

**Figure 1:**
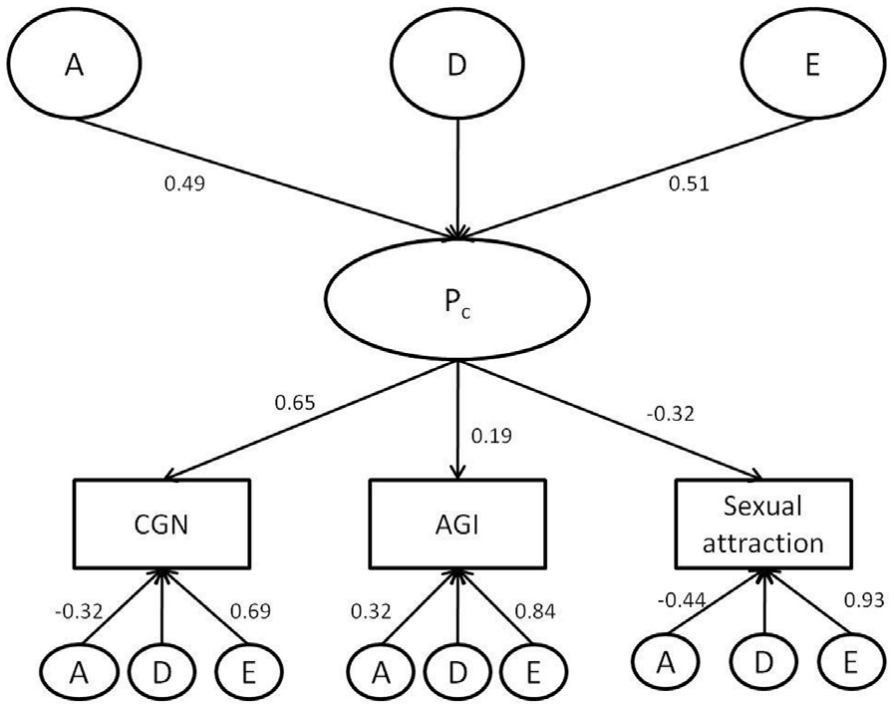
Best fitting common pathway model. The figure shows standardized parameter estimates for the path coefficients of the common pathway model, selected as the most appropriate depiction of the data. The squares of the path coefficients provide an estimate of the variance explained by common and specific genetic and environmental components.

When comparing the independent and common pathway models with the Cholesky model, the common pathway model was found to offer the most suitable explanation of the data with the lowest value of AIC at 3871.1 and the lowest BIC at −22609.7 (Table 5). This common pathway model explains the variance in each variable in terms of unique A, D and E contributions as well as a contribution from the “common sexual orientation phenotype” (P_c_). The parameter estimates derived from the common pathway model are shown in Figure 1. To obtain the contribution that P_c_ and unique A, D and E make to the variance in a trait, squares of the path coefficient are taken. Thus, the model postulates the existence of an underlying sexual orientation phenotype (P_c_) with a heritability 24% (0.49^2^) that chiefly explains the co-occurrence of CGT, AGI and sexual attraction. For CGT Pc accounts for 43% (0.65^2^) of the variation, for AGI it accounts for 4% (0.19^2^) and for sexual attraction it accounts for 11% (−0.32^2^) of the variation. The heritability of the variation in the phenotype that is not accounted for by Pc is 10% (−0.32^2^) for CGT, 10% (0.32^2^) for AGI and 19% (0.44^2^) for sexual attraction. No influence of D on the variation of Pc and the phenotypes could be detected. Overall, these results suggest that the common sexual orientation phenotype does not account for significant variation in AGI in this model.

**Table 5:** Multivariate analysis of three models showing change in model fit (*χ*2) and degrees of freedom (*df*) when specified parameters are dropped from full ADE model (best fitting models in bold).

	Model	*df*	AIC	BIC	−2LL
Cholesky	ADE	9008	3875.84	−22588.23	21891.84
Independent	ADE	9011	3876.25	−22596.21	21898.25
***Common***	***ADE***	***9015***	***3871.08***	***−22609.67***	***21901.08***

AIC = Akaike Information Criterion. AIC describes the model with best goodness-of-fit combined with parsimony. BIC = Bayesian Information Criterion. −2LL = likelihood ratio chi-square test as a measure of goodness of fit.

## Discussion

Our results show that sexual attraction and CGT are influenced by genetic factors (accounting for 25% and 32% of the variance respectively). Genetic contributions as estimated in the univariate analyses had a much weaker impact on AGI (11%). The effect of non-shared environmental factors (including measurement error) on all traits was large. However, there was no effect of the shared family environment on any trait.

These findings are broadly consistent with previous population-level twin studies demonstrating a heritable basis to male and female sexual orientation. The heritability estimates reported here for female sexual attractions were larger than those reported by Bailey et al. [19] for sexual attraction components (8%). For attraction, we found no effect of the shared environment in contrast to Bailey et al. [19] who reported an estimate of 41%. Langstrom et al. [21] reported shared environmental effects of same-sex sexual behavior of 17%. Kendler et al. [20] did not separate their analysis by sex so we cannot compare the findings. Finally, our genetic estimates were lower than those reported by Kirk et al. [22] who attempted to model two components of sexual orientation - sexual attraction and sexual experience - and reported estimates for females between 50 and 60%. Whilst Kirk et al. [22] did use attractions in their study they supplemented these with the measures “attitudes to homosexual sex” and “lifetime same-sex partners” (from a range in an extensive sexual orientation questionnaire) which are not directly comparable to measure used here.

The effect of E on all traits was large. E includes phenotypic variation accounted for by non-shared environment and measurement error. A variety of sources can cause measurement error, including inadequate or imprecise assessment instruments and phenotype description, and a variety of response styles, specifically acquiescence, disacquiescence, extreme response, midpoint responding, and noncontingent responding [52]. The twin modelling approach used in this study does not allow separation of the two sources, hence, quantification of the influence of measurement error is impossible. It is therefore likely that some inconsistency between our heritability estimates for sexual attraction compared to previous work might be due to different usage of Kinsey-type scales for measuring trait sexual orientation. Here we used a 5-item measure; Bailey et al. [19] used the full 7-item Kinsey-scale; Langstrom et al. [21] used number of same-sex partners; and Kendler et al. [20] employed a single item with three response choices (heterosexual, bisexual, and homosexual in attractions). If we compare our data to Bailey et al. (both studies focused on attractions and using what approximate traditional Kinsey-type scales), it is possible that the relatively small difference in response options between the two studies (that is, a difference of 2) contributed to the differing heritability estimates for sexual attraction. Parameter estimates for sexual orientation might be unusually sensitive to the range of items used to assess the trait and thus future researchers should be mindful of the utilizing psychometrically robust scales.

Consistent with several studies the highest heritability was found for CGT (32%) [19], [30]. CGT seems to be the highest heritable correlate of sexual orientation reported thus far, and furthermore lies in the region of the *h* ^2^ estimates generally reported for sexual orientation (measured both behaviorally and psychologically). This adds further support to the notion that CGT may be the main heritable component or endophenotype of sexual orientation [53], [54]. However, genetic effects for AGI were negligible compared to previous work and we are less confident about the validity of AGI as a robust correlate of sexual orientation [19]. The inter-correlations with other measures of sexual orientation were low for AGI compared to CGT and showed little variability. Compared with CGT items which capture sex-typed behavior, interests and identity, AGI comprises identity items only and thus has restricted psychometric precision. Other documented indices of gender identity such as “gender diagnosticity” e.g. [27] should be the focus of future twin studies if suitable short-form scales can be developed. As with attraction, the inconsistencies between the studies might be attributed to the number of items in the measures used. Our measure of CGT comprised four items and Bailey et al. 's [19] five items used to check the reliability of self- vs. other-report of their 24-item measure . The relative comparability here (a difference between the two studies of only 1 item) provides some confidence for the validity of heritability estimates reported by both. However, their measure of AGI comprised seven items and ours only four (with relatively low internal consistency) which may explain the differences between studies for this particular measure.

The results from the multivariate analyses presented here provide evidence of a genetic overlap between CGT and sexual attraction but less for AGI. We detected a common latent phenotype with a heritability of 24% underlying sexual orientation, CGT and AGI as well as moderate phenotype-specific additive genetic factors and large phenotype-specific non-shared environmental factor loading on these traits. These data are supportive of those from Bailey et al. [19] who also found that a common pathway ACE model best fitted the available data. Both studies support the notion that showed that genetic and non-shared environmental factors markedly contributed to the covariation among the measures, with all three measured variables (sexual attraction, CGT and AGI) being good indicators of an underlying latent factor. Overall, the present results support previous non-twin evidence for the existence of an intermediate phenotype for sexual orientation, such as for example “sex-atypicality” [54]. A likely candidate for this latent phenotype is prenatal androgen exposure which shapes variations in gender nonconforming behavior and sexual orientation and the developmental coupling between them [1], [33], [35]. Nevertheless, speculation about origins of this putative sex hormone-related phenotype is limited by two candidate gene studies of male sexual orientation both producing null results: one for the androgen receptor [55] and another for aromatase [56]. However, the absence of such associations in men does not imply a similar null result in women.

Insofar as our genetic estimates are additive, these data do not suggest a major role for epistatic or dominant allelic effects. Our results are also silent on the sources of non-shared environmental effects. However, what is clear from several twin studies, including the present, is that shared factors such as the home environment and parenting styles have little impact on human sexual orientation. Nevertheless, as we cannot be certain that our measures, particularly AGI, were robust (given the sizable loading of a common non-shared environmental factor on each trait) there is a necessary degree of imprecision to our parameter estimates and the model fitting results should be treated with caution. Future studies should be particularly careful in using measures of AGI. Several other limitations weaken overly strong conclusions from the present study. The response rate was lower than three other large twin studies (49%, compared to 53.8% in Bailey et al. [19] 60% in Kendler et al. [20]; and 59.6% in Langstrom et al. [21]) although it is not clear how this could systematically bias the parameter estimates reported [57]. Also, the response rate here is in fact comparable to other epidemiological surveys of female sexual behavior [58], [59]. The representativeness of our twin sample also diminishes any putative selection biases, as shown by a large comparative study demonstrating that our twin population is very similar to singletons on a wide range of common health and lifestyle factors [60]. A comparison of the sample characteristics in Table 1 show that the MZ and DZ twins did not differ significantly on most demographic variables, arguing against the tendency for MZ to be more alike possibly due to shared upbringing. The comparably low internal consistencies for CGT and AGI may further reflect the heterogeneous nature of the constructs, suggesting that more items are needed to capture the range of manifestations of the constructs. We also used retrospective measures of CGT which may be influenced by recall biases. However, prospective studies confirm the predictive psychometric validity of measures of CGT that are comparable to the one used here as do studies of maternal reports of proband-recalled CGT and studies of childhood home videos [23], [33].

Due to our considerably large sample size we had enough power to detect a rather small contribution of non-additive genetic factors, had it been present (1,800 twin pairs are needed to reject an AE model with a power of 80% when an ADE model is the true model, with respective contributions of additive genetics effects = 0.50, dominant genetic effects = 0.30 and non-shared environmental effects = 0.20) [48]. Nonetheless, there remained insufficient numbers of non-heterosexual participants to guarantee a high degree of statistical power in the genetic and environmental analyses. This is a well-known problem, as sexual orientation-related data are notoriously skewed [21].

In summary, we found genetic influences on female sexual orientation as measured via attractions and on CGT (a key developmental correlate of sexual orientation). A moderate effect of a common latent phenotype suggests that there are some overlapping mechanisms which may be responsible for sexual orientation. However, stronger conclusions are not warranted at this stage because of substantial measurement error. Future research efforts should focus on “sex-atypicality” as a possible intermediate phenotype for trait sexual orientation which may be more amenable to gene-mapping approaches.
